# Histopathological and risk factor analyses of oral potentially malignant disorders and oral cancer in a proactive screening in northeastern Thailand

**DOI:** 10.1186/s12903-022-02646-9

**Published:** 2022-12-16

**Authors:** Boworn Klongnoi, Vanvisa Sresumatchai, Harin Clypuing, Angkana Wisutthajaree, Jintana Pankam, Natchalee Srimaneekarn, Binit Shrestha, Siribang-on Piboonniyom Khovidhunkit

**Affiliations:** 1 Department of Oral and Maxillofacial Surgery, Faculty of Dentistry, Mahidol University, Bangkok, Thailand; 2 Department of Family Health, Faculty of Public Health, Mahidol University, Bangkok, Thailand; 3 Dental Department, Maharat Nakorn Ratchasima Hospital, Nakhon Ratchasima, Thailand; 4 Employee of the Development of Disease Management Model for Oral Cancer with an Integration Network of Screening, Surveillance, and Treatment in North East Health District Grant, Faculty of Dentistry, Mahidol University, Bangkok, Thailand; 5 Department of Anatomy, Faculty of Dentistry, Mahidol University, Bangkok, Thailand; 6 Maxillofacial Prosthetic Unit, Department of Prosthodontics, Faculty of Dentistry, Mahidol University, Bangkok, Thailand; 7 Department of Advanced General Dentistry, Faculty of Dentistry, Mahidol University, 6 Yothi Rd., Rajthewee, Bangkok, 10400 Thailand

**Keywords:** Oral potentially malignant disorders, Oral cancer, Thais, Screening

## Abstract

**Background:**

Lip and oral cavity cancer has been reported as the 10th most common cancer in Thailand. Recently, a screening program for oral potentially malignant disorders (OPMDs) and oral cancer was conducted in the northeastern Thailand which took into consideration a total of 371,911 people who resided in the provinces of Buriram, Chaiyaphum, Nakhon Ratchasima, and Surin.

**Methods:**

A total of 330,914 subjects were consecutively screened for risk factors of oral cancer by village health volunteers (VHVs) using a questionnaire (S1). Then, 186,710 subjects with one or more risk factors for oral cancer were referred for oral screening by dental auxiliaries or dentists at sub-district level hospitals (S2) where 86,941 subjects were subsequently screened. Afterwards, 1576 subjects with suspicious oral lesions for OPMDs or oral cancer attended local hospitals for further investigation and treatment. Oral medicine specialists, oral surgeons, and local dentists at the district level hospitals performed biopsies and the samples were sent for histopathological analysis. The objectives of the study were to report the histopathology findings from the biopsies obtained from these subjects and the associated risk factors.

**Results:**

Out of 427 subjects who received biopsies, complete diagnostic results were obtained from 409 patients (462 specimens). The 5 most common histopathological results from these specimens were mild epithelial dysplasia (27.3%), fibroepithelial hyperplasia (14.5%), oral lichen planus/oral lichenoid reactions (11.5%), moderate epithelial dysplasia (8%), and acanthosis with or without hyperkeratosis (5%). Oral squamous cell carcinoma was detected in 14 subjects and 11 other forms of oral cancer were revealed. Among the analyzed risk factors, habitual betel quid chewing was established as a statistically significant risk factor associated with OPMDs and oral cancer.

**Conclusion:**

The most frequently observed histopathological results of clinically suspected oral cancer and OPMDs included mild epithelial dysplasia, fibroepithelial hyperplasia, oral lichen planus/oral lichenoid reactions, moderate epithelial dysplasia, and acanthosis with or without hyperkeratosis. Betel quid chewing habit was found to be associated with OPMDs and oral cancer.

**Supplementary Information:**

The online version contains supplementary material available at 10.1186/s12903-022-02646-9.

## Background

In 2020, the International Agency for Research on Cancer, WHO, estimated that the number of new cancer cases was 190,636 in Thailand, and lip and oral cavity cancer was the 9th most common cancer [1]. A study on the epidemiology of oral cancer in Asia reported the prevalence to be especially high in the southern and southeastern countries [2]. Habitual betel quid chewing, varying patterns of tobacco use, and alcohol consumption are pivotal risk factors that predispose the population to oral cancer. Furthermore, most rural Asians rely on agriculture, which makes them prone to extended durations of sun exposure, especially when working in the fields that may lead to lip and skin cancers. From the study by Krishna and colleagues, the mean age of occurrence of oral cancer is usually between 51 and 55 years in most countries, with tongue being the most common site (42%), followed by buccal mucosa (29.7%) and gingiva (19.8%) [2]. Despite great strides in the fields of diagnosis and treatment, the 5-year survival rate of oral cancer is still low. In rural India, relative survival rates were 38% and 42% for tongue and other areas of mouth, respectively [3].

Screening of oral cancer has been performed in many countries [4, 5]. From a study carried out in Kerala, India, sustained reduction in oral cancer mortality was observed during a 15-year follow up, with larger reductions noted in those returning for repeated rounds of screening. These results have been encouraging towards the introduction of population-based screening programs targeting users of tobacco or alcohol or both in high-incidence countries [5]. Although the 12% reduction in oral cancer mortality in all individuals did not reach statistical significance, there was a 24% reduction in oral cancer mortality (95% confidence interval [CI] 3–40%) in users of tobacco and/or alcohol [4]. Oral cancer screening in people with high risk factors has also been carried out in Taiwan [6]. A total of 2,334,299 individuals aged ≥ 18 years old with cigarette smoking or betel quid chewing habits were enrolled in the study. Subjects were visually screened for oral potentially malignant disorders (OPMDs) or oral malignancy by dentists or physicians and referred to the hospitals for histopathological diagnosis. Subjects who were screened negative were re-examined after 2 years. The results showed that the relative risk of advanced oral cancer for the screened group versus non-screened group was 0.62 (95% CI 0.59–0.64). The relative risk of death from oral cancer was 0.53 (95% CI 0.51–0.56) as a result of screening compared with the expected risk of oral cancer deaths in the absence of screening. In a cross-sectional study, a total of 762 individuals who attended Basic Health Units in Fernandόpolis city, Brazil were screened for oral cancer. Following subsequent screenings, benign lesions were diagnosed in 56 individuals (77.34%) and only 1 individual (0.13%) was confirmed with malignant lesion [7]. The low incidence rate indicated that oral cancer prevention methodologies needed to be improved and should focus on screening of oral cancer or OPMDs in high risk individuals to increase their effectiveness.

Frequently, oral cancer is preceded by OPMDs. Although the majority of these disorders are asymptomatic in the early stages, they can be detected by dental practitioners during routine oral examination. Early detection of these disorders can be beneficial to reduce cancer related morbidity and mortality [8]. OPMDs are lesions or conditions with a predisposition to malignant transformation [9], and include oral leukoplakia, oral erythroplakia, proliferative verrucous leukoplakia, oral lichen planus, oral lichenoid lesions, oral submucous fibrosis, palatal lesions in reverse smokers, lupus erythematosus, dyskeratosis congenita, actinic cheilitis, and chronic graft-versus-host disease [10].

Oral cancer and potentially malignant disorder screening programs have been previously conducted in some regions of Thailand. In a study at the dental department of Chaiyaphum provincial hospital in the northeastern region, the incidence of OPMDs and oral cancer among 379 patients was 2.9% and 0.3%, respectively [11]. Higher incidences were found in females and increased with old age. Factor significantly associated with OPMDs was betel quid chewing habit (OR = 27.68, 95% CI 6.96–110.98). Similarly, a cross sectional descriptive study was conducted in Roi Et province to examine the prevalence of oral premalignant lesions and associated factors [12]. The data were collected using a questionnaire and by reviewing patient’s medical records. Totally, 2300 subjects over 40 years of age were included. Out of 2300 subjects, 102 subjects (3.8%) presented with oral premalignant lesions. The oral premalignant lesions detected were oral leukoplakia (34 cases, 1.5%), oral lichen planus (33 cases, 1.4%), erythroplakia (19 cases, 0.8%), and oral submucous fibrosis (1 case, 0.04%). Strong association was also found between risk factors, such as betel quid chewing, smoking and alcohol consumption, and the presence of oral premalignant lesions [12].

Screening for oral cancer and potentially malignant disorders in selected group of subjects with high risk for oral cancer has shown effectiveness towards early detection of cancer; moreover, attaining a definitive histopathological diagnosis and understanding the associated risk factors are equally important. Therefore, the objectives of this study were to report the histopathology results of subjects who attended the screening for OPMDs and oral cancer in the northeastern region of Thailand and to investigate the association between the risk factors of oral cancer and the presence of OPMDs and oral cancer.

## Methods

### Subject selection

**Fig. 1:**
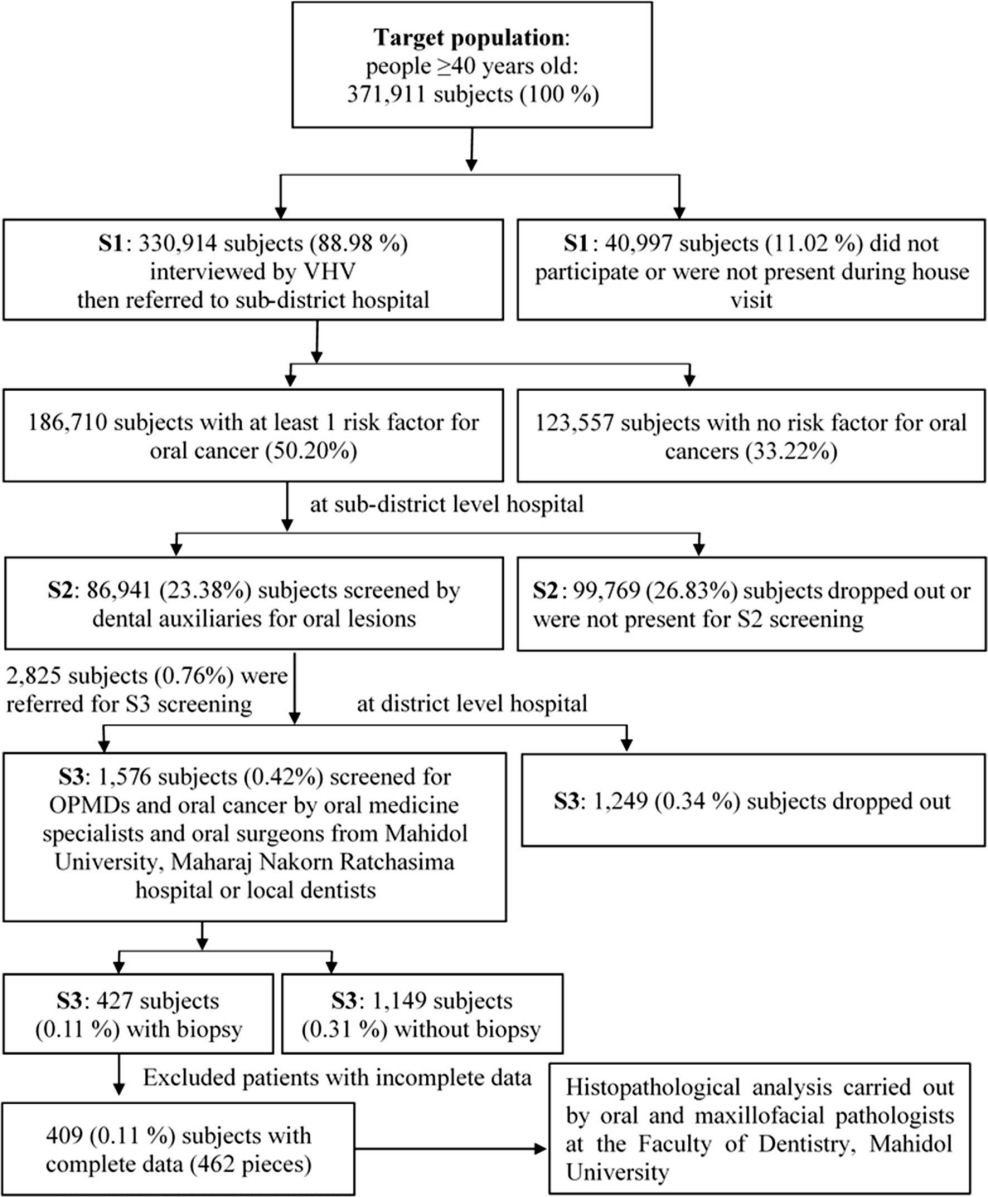
Schematic representation of the subject recruitment and conceptual framework

This study was a part of the project entitled “Development of Disease Management Model for Oral Cancer with an Integration Network of Screening, Surveillance, and Treatment in Northeast Health District” conducted for screening of oral cancer and OPMDs in the northeastern part of Thailand. Between May 2019 and August 2020, individuals aged ≥ 40 years old who lived in the 4 northeastern provinces of Buriram, Chaiyaphum, Nakhon Ratchasima, and Surin were selected. A target population of 371,911 subjects was estimated from the registry of the Ministry of Public Health. Three steps of cancer screening including Screening 1 (S1), Screening 2 (S2), and Screening 3 (S3) were conducted (Fig. 1).

- In S1 level, the subjects were interviewed at their homes by village health volunteers (VHVs) using a standardized objective questionnaire to assess their risk behavior. The inclusion criteria for the screening were age ≥ 40 years old, presented with current or history of smoking or alcohol consumptions, betel quid chewing habits, smokeless tobacco use, working in the fields with strong sunlight, presence of chronic oral ulcers or irritation due to sharp tooth, dental caries, dental calculus, periodontal disease or ill-fitting removable denture(s). After the S1 screening, subjects who had at least 1 risk factor were referred for S2 screening at sub-district level hospitals.
- In S2 level, the referred subjects were visually screened intra-orally for OPMDs or oral cancer or other benign oral lesions by dental auxiliaries or local dentists, who had received prior formal education. Before the initiation of the study, they had also received specialized training under close supervision by BK and SPK. Details regarding the risk factors were reverified and transferred to the Oral Cancer Screening Program developed by the Faculty of Public Health, Mahidol University. Subjects who had these lesions were referred to the district or provincial hospital for S3.
- In S3 level, subjects with suspicious oral lesions were examined by local dentists, specialists in oral medicine or oral and maxillofacial surgery from the Faculty of Dentistry, Mahidol University or Maharat Nakhon Ratchasima Hospital. Examination of the oral cavity was carried out under ample light illumination from a dental unit followed by digital palpation of the lesions. Photographs were taken for reference. Inclusion criteria for the biopsy were suspicious OPMDs, oral cancer, or other benign oral lesions in subjects who agreed to undergo a biopsy. Exclusion criteria were known cases of oral cancer or OPMDs, severe systemic diseases such as cancers elsewhere, severe blood diseases, uncontrolled diabetes, bleeding tendency, or severe cardiac problems. Subjects who had problems in communication or those who refused to participate in the project were also excluded. The pathological diagnosis of the biopsied lesions was carried out by a group of board-certified oral pathologists. OPMDs were diagnosed according to the WHO recommendations [10]. The definitive histopathological diagnosis of the OPMDs included acanthosis with or without hyperkeratosis, mild, moderate or severe epithelial dysplasia, oral lichen planus, oral lichenoid lesions, oral lupus erythematosus, and oral submucous fibrosis.

The characteristics of oral lesions were recorded in the S3 form then the data were transferred to the program (http://ocphmodel.dt.mahidol.ac.th/oralcancerr9/index.php) developed by the Faculty of Public Health, Mahidol University. Individuals with confirmed cancerous lesions were treated with surgical removal of the lesion along with adjunctive or supportive therapies. For the follow-up programs, individuals with OPMDs and non-OPMDs were recalled bi-yearly at S3 level for continual examination and treatment as required. Individuals with no lesion, or without a confirmatory diagnosis were followed up at S2 level at 2 weeks, 1 month, and 2 months. [13].

### Sample size calculation

Sample size was calculated using the prevalence of oral precancerous lesions detected in a study in Thailand (2014) by Juntanong et al. which was 4.4% [12]. The following formula was applied with 95% CI and d = 0.05.

$${\text{n}} = {\text{Z}}^{2} {{\left( {{\text{PQ}}} \right)} \mathord{\left/ {\vphantom {{\left( {{\text{PQ}}} \right)} {\left( {{\text{dP}}} \right)^{2} }}} \right. \kern-\nulldelimiterspace} {\left( {{\text{dP}}} \right)^{2} }}$$

After the calculation, 33,388 subjects were initially considered; however, with a 40% non-response rate, the minimum number of subjects required for this study was calculated as 46,743.

### Statistical analysis

Descriptive statistical analysis was presented as frequencies, percentages, and mean ± SD. Pearson Chi-Square or Fisher's Extract Test was used as appropriate. Statistical significance in the final model was set at *p* < 0.05.

To determine the relationship between the risk factors and cancer/OPMDs, subjects were categorized into non-OPMDs/oral cancer and OPMDs/oral cancer groups. Subjects with more than one lesion were included in the OPMDs/oral cancer group. Multiple logistic regression was performed using all variables with probability values < 0.2 following bivariate analysis. All analyses were performed using the SPSS (version 18, license:spss.Mahidol).

## Results

### Participant recruitment

The recruitment strategy of the subjects is demonstrated in Fig. 1. Firstly, the target population selected for the screening of OPMDs and oral cancer was set at 371,911 subjects according to the registry of people who were ≥ 40 years old residing in the provinces of Buriram, Chaiyaphum, Nakhon Ratchasima, and Surin. A total of 25,338 VHVs were involved in the S1 screening, and approximately half of the target population (186,710 subjects) were identified with at least one risk factor for oral cancer. Approximately half of these subjects (86,941 subjects) underwent the S2 screening by 131 dental auxiliaries or 53 local dentists. A total of 2825 subjects were then referred for further examination at the district level hospitals. The S3 screening was conducted by 53 local dentists and 9 specialists. Out of 1576 subjects who participated, 427 subjects agreed to undergo biopsy for histopathology analysis. Finally, after the exclusion of the subjects with incomplete data, 409 individuals (462 biopsy specimens, i.e. ≥ 1 lesion may be present in an individual) were analyzed for the prevalence of OPMDs, oral cancer, or other non-OPMDs/oral cancer lesions. All individuals with cancerous lesions received proper treatment, whereas individuals with OPMDs were monitored by local dentists and specialists under the surveillance program every 3–6 months at S3 level. Individuals without OPMDs or no identifiable lesions were categorized as low risk and followed up by dental auxiliaries at S2 level every 6 months.

### Subjects’ characteristics

The majority of study subjects were female (73%), and the most prevalent age was between 60 and 79 years old (Table 1). Among the male subjects, a large number of them were smokers, drinkers, or had been regularly exposed to long durations of sunlight. On the other hand, female subjects habitually chewed betel quid, consumed smokeless tobacco, or were second hand smokers (Table 1). The prevalence of OPMDs was higher in females while that of oral cancer and non-OPMDs/oral cancer was higher in males.

**Table 1:** Characteristic of study subjects (n = 409)

Characteristics	Male		Female		All	
	n	%	n	%	n	%
Sex	111	27.14	298	72.86	409	100.00
Age (years)						
40–49	8	7.21	11	3.69	19	4.65
50–59	24	21.62	54	18.12	78	19.07
60–69	48	43.24	104	34.90	152	37.16
70–79	26	23.42	104	34.90	130	31.78
80–89	5	4.50	21	7.05	26	6.36
≥ 90	0	0.00	4	1.34	4	0.98
Mean ± SD	64.30 ± 9.17		66.10 ± 9.61		66.34 ± 9.57	
Minimum–Maximum	42–88		42–94		42–94	
Smoking						
Never	46	41.44	289	96.98	335	81.91
Smoker	47	42.34	6	2.01	53	12.96
Ex-smoker	18	16.22	3	1.01	21	5.13
N/A	0	0.00	0	0.00	0	0.00
Smokeless tobacco						
Never	91	81.98	235	78.86	326	79.71
Smoker	12	10.81	55	18.46	67	16.38
Ex-smoker	5	4.50	6	2.01	11	2.69
N/A	3	2.70	2	0.67	5	1.22
Secondhand smoker						
No	72	64.86	201	67.45	273	66.99
Yes	27	24.32	86	28.86	113	27.43
N/A	12	10.81	11	3.69	23	5.58
Alcohol drinking						
Never	48	43.24	240	80.54	288	70.42
Alcohol drinker	43	38.74	40	13.42	83	20.29
Ex-alcohol drinker	19	17.12	16	5.37	35	8.56
N/A	1	0.90	2	0.67	3	0.73
Betel quid chewing						
Never	98	88.29	113	37.92	211	51.59
Chewer	10	9.01	167	56.04	177	43.28
Ex-chewer	2	1.80	18	6.04	20	4.89
N/A	1	0.90	0	0.00	1	0.24
Working in sunlight more than 4 days a week						
No	34	30.63	153	51.34	187	45.72
Yes	75	67.57	142	47.65	217	53.06
N/A	2	1.80	3	1.00	5	1.22
History of head and neck cancer						
No	97	87.39	283	94.97	380	92.91
Yes	9	8.11	15	5.03	24	5.87
N/A	5	4.50	0	0.00	5	1.22
Oral lesions						
Cancerous lesions	9	8.11	13	4.36	22	5.38
OPMD	53	47.75	183	61.41	236	57.70
Non-OPMD/oral cancer	49	44.14	102	34.23	151	36.92

*OPMDs* oral potentially malignant disorders, *SD* standard deviation

### Distribution of oral lesions according to clinical diagnosis

In 409 subjects, 462 lesions were clinically identified. All lesions were categorized into 3 groups including oral cancer, OPMDs, and other non-OPMDs/oral cancer (Table 2). Out of 462 lesions, squamous cell carcinoma was found in 3% and verrucous carcinoma in 1.3%. The five other types of oral cancerous lesions were clear cell odontogenic carcinoma, mucoepidermoid carcinoma, adenoid cystic carcinoma, lymphoma, and basal cell carcinoma.

**Table 2:** Distribution of oral lesions according to clinical diagnosis

Lesion	n = 462** (specimens)	%
*Cancerous lesions*	25	5.41
Oral squamous cell carcinoma	14	3.03
Oral verrucous carcinoma	6	1.30
Other oral cancers	5	1.08
OPMDs*	264	57.14
Oral leukoplakia	171	37.01
Oral lichan planus/oral lichenoid lesions	53	11.47
Oral erythroplakia	17	3.68
Oral lupus erythematosus	6	1.30
Actinic cheilitis	8	1.73
Oral submucous fibrosis	2	0.43
Other OPMDs	7	1.52
*Non-OPMDs/oral cancer*	173	37.45
Fibroma	82	17.75
Squamous papilloma	19	4.11
Pyogenic granuloma	10	2.16
Inflamed mucosa	9	1.95
Frictional keratosis	9	1.95
Hyperplastic candidiasis	7	1.52
Others	37	8.01

*OPMDs oral potentially malignant disorders

^**^≥ 1 lesion may be present in an individual

OPMDs were found in 57.1% of all oral lesions. Overall, oral leukoplakia was clinically diagnosed in 37.0% and oral erythroplakia in 3.7%. Interestingly, 11.5% of the specimens were diagnosed with oral lichen planus or oral lichenoid lesions, and they were the third most common pathological diagnosis encountered.

Regarding non-OPMDs/oral cancer lesions, reactive oral lesions including fibroma, squamous papilloma, and pyogenic granuloma were often discovered. The distribution of the oral lesions according to clinical diagnosis is summarized in Table 2.

### Distribution of oral lesions according to histopathological diagnosis

**Fig. 2:**
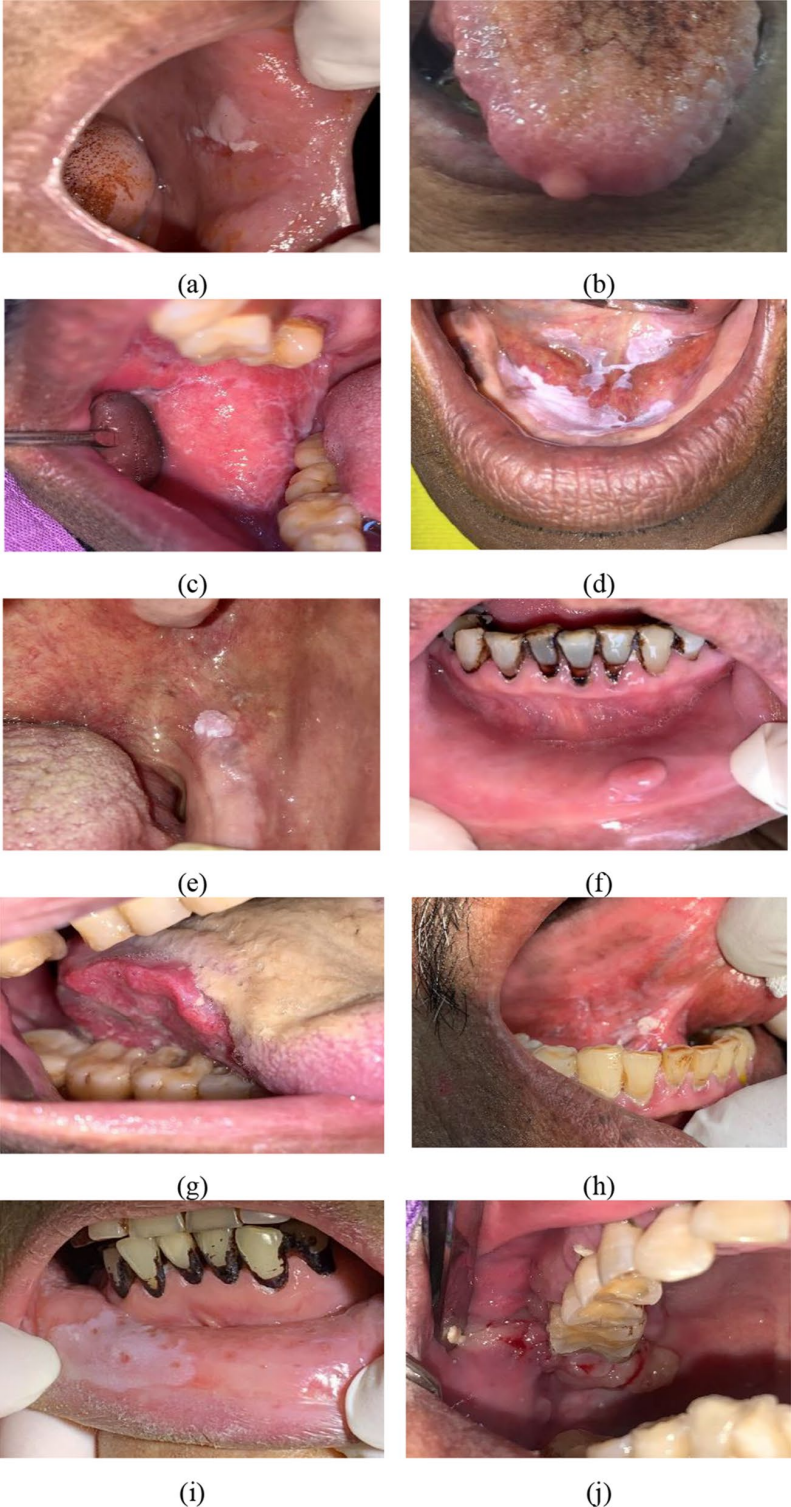
Representative clinical pictures of the 10 most common oral lesions according to histopathological diagnosis. **a** Mild epithelial dysplasia; **b** fibrous hyperplasia; **c** oral lichen planus; **d** moderate epithelial dysplasia; **e** squamous papilloma; **f** giant cell fibroma; **g** squamous cell carcinoma; **h** severe epithelial dysplasia; **i** acanthosis with hyperkeratosis; **j** pyogenic granuloma

Among the 462 lesions clinically identified and biopsied, the 10 most common histopathological diagnosis were mild epithelial dysplasia (27.3%), fibro-epithelial hyperplasia (14.5%), oral lichen planus/lichenoid lesions (11.5%), moderate epithelial dysplasia (8%), acanthosis with or without hyperkeratosis (5%), squamous papilloma (4.1%), severe epithelial dysplasia (3.7%), giant cell fibroma (3.3%), squamous cell carcinoma (3%), and pyogenic granuloma (2.2%) (Table 3). Figure 2 demonstrates representative clinical pictures of 10 most common oral lesions according to histopathological diagnosis. Additional file 1 demonstrates the characteristics of subjects and their risk factors of oral cancer according to types of oral lesions diagnosed by histopathology results.

**Table 3:** Distribution of oral lesions according to histopathological diagnosis

Lesion	n = 462 (specimens)	%
Mild epithelial dysplasia	126	27.27
Fibro-epithelial hyperplasia	67	14.50
Oral lichen planus/oral lichenoid lesions	53	11.47
Moderate epithelial dysplasia	37	8.01
Acanthosis with or without hyperkeratosis	23	4.98
Squamous papilloma	19	4.11
Severe epithelial dysplasia	17	3.68
Giant cell fibroma	15	3.25
Oral squamous cell carcinoma	14	3.03
Pyogenic granuloma	10	2.16
Inflamed mucosa	9	1.95
Hyperplastic candidiasis	7	1.52
Oral lupus erythematosus	6	1.30
Oral verrucous carcinoma	6	1.30
Oral submucous fibrosis	2	0.43
Clear cell odontogenic carcinoma	1	0.22
Mucoepidermoid carcinoma	1	0.22
Adenoid cystic carcinoma	1	0.22
Oral lymphoma	1	0.22
Basal cell carcinoma	1	0.22
Others	46	9.96

### Bivariate analysis of risk factors of oral cancer and types of oral lesions following definitive diagnosis

To evaluate the association between risk factors of oral cancer and types of oral lesions, bivariate analysis of the risk factors of oral cancer and types of oral lesions was performed (Table 4). The subjects were categorized into two groups, namely non-OPMDs/oral cancer and OPMDs/oral cancer. The mean age of occurrence between the 2 groups were not significantly different. Subjects in the age group 60–79 years with a previous history or currently using smokeless tobacco and/or betel quid chewing habit were more likely to have OPMDs/oral cancer (Table 4). On the other hand, non-OPMDs/oral cancer lesions were observed more in subjects who had a previous history of head and neck cancer.

**Table 4:** Characteristics and risk factors of study subjects according to the types of oral lesions

Characteristics	Non-OPMDs/cancer		OPMDs/cancer		Total		OR (95% CI)	*p* value
	n	%	n	%	n	%		
*Sex (n* = *409)*								
Male	49	32.45	62	24.03	111	27.14	Ref.	
Female	102	67.55	196	75.97	298	72.86	1.519 (0.974–2.369)	0.065
*Age (year) (n* = *409)*								
40–59	45	29.80	52	20.16	97	23.72	Ref.	
60–79	96	63.58	186	72.09	282	68.95	1.677 (1.049–2.680)	0.031
≥ 80	10	6.62	20	7.75	30	7.33	1.731 (0.734–4.080)	0.210
Mean ± SD	64.64 ± 9.59		67.34 ± 9.43		66.34 ± 9.57			
Minimum	42		43		42			
Maximum	89		94		94			
*Smoking (n* = *409)*								
Never	122	80.79	213	82.56	335	81.90	Ref.	
Smoker/ex-smoker	29	19.21	45	17.44	74	18.10	0.889 (0.530–1.491)	0.655
*Smokeless tobacco (n* = *404)*								
Never	132	88.00	194	76.38	326	80.69	Ref.	
Smoker/ex-smoker	18	12.00	60	23.62	78	19.31	2.268 (1.281–4.016)	0.005
*Secondhand smoker (n* = *386)*								
No	104	73.24	169	69.26	273	70.73	Ref.	
Yes	38	26.76	75	30.73	113	29.27	1.215 (0.766–1.925)	0.408
*Alcohol drinking (n* = *406)*								
Never	100	66.67	188	73.44	288	70.94	Ref.	
Drinker/ex-drinker	50	33.33	68	26.56	118	29.06	0.723 (0.467–1.121)	0.148
*Betel quid chewing (n* = *408)*								
Never	104	69.33	107	41.47	211	51.72	Ref.	
Chewer/ex-chewer	46	30.67	151	58.53	197	48.28	3.191 (2.083–4.887)	< 0.001
*Working in sunlight (n* = *404)*								
No	67	45.27	120	46.88	187	46.29	Ref.	
Yes	81	54.73	136	53.12	217	53.71	0.937 (0.624–1.407)	0.755
*History of head and neck cancer (n* = *404)*								
No	133	90.48	247	96.11	380	94.06	Ref.	
Yes	14	9.52	10	3.89	24	5.94	0.385 (0.166–0.890)	0.026

*OPMD* oral potentially malignant disorders, *SD* standard deviation

### Association of risk factors and types of oral lesion

After bivariate analysis, six variables with *p* value less than 0.2 were included in the multiple logistic regression analysis and the results are presented in Table 5. Multivariable analysis of the risk factors showed betel quid chewing as a significant risk factor for oral cancer and OPMDs (Adjusted OR = 2.93, 95% CI 1.75–4.88) (Table 5).

**Table 5:** Multivariable analysis of risk factors associated with OPMDs and oral cancer

Variables	Odds ratio (95% CI)^a^	*p* value
*Sex*		
Male	1	0.348
Female	0.768 (0.442–1.334)	
*Age (year)*		
40–59	1	
60–79	1.202 (0.721–2.003)	0.480
≥ 80	0.975 (0.388–2.451)	0.957
*Smokeless tobacco*		
Never	1	0.113
Smoker	1.636 (0.890–3.009)	
*Alcohol drinking*		
Never	1	0.234
Drinker	0.738 (0.448–1.217)	
*Betel quid chewing*		
Never	1	< 0.001*
Chewer	2.925 (1.753–4.880)	
*History of head and neck cancer*		
No	1	0.103
Yes	0.478 (0.196–1.162)	

^*^Statistically significant

^a^Adjusted OR (95% CI)

## Discussion

This study was aimed to investigate the type and frequency of OPMDs, oral cancer, and other non-OPMDs/oral cancer lesions with confirmed histopathological diagnosis in the subjects who attended oral cancer screening in the northeastern region of Thailand.

This study was one of the largest proactive OPMDs/oral cancer screening in Thailand which targeted 371,911 individuals from 4 provinces including Buriram, Chaiyaphum, Nakhon Ratchasima, and Surin. As shown in Fig. 1, the VHVs interviewed 330,914 subjects and approximately half of them were found to have at least one risk factor for OPMDs or oral cancer. This signified that a large number of individuals were at risk of developing oral cancer in the future. In the S2 screening, almost 90,000 subjects participated, and there were approximately 3000 subjects (one in 30) identified with potential oral lesions. The dental auxiliaries who partook in this study had been trained and calibrated to detect and refer suspicious oral abnormalities for further screening by dentists or dental specialists. Subsequently, 1576 subjects or approximately half underwent the S3 screening at the district hospitals, where the oral medicine specialists and oral and maxillofacial surgeons readily performed biopsies of suspected lesions. The remainder who did not undergo biopsy either received medication for the treatment, denied treatment, had contraindications for biopsy, or were appointed for further follow up as explained in our previous study [13]. Concurrently, all subjects were also encouraged to quit or reduce the risk factors for oral cancer. Finally, 427 subjects underwent biopsies (Fig. 1) and 258 subjects were diagnosed with OPMDs or oral cancer (Table 1). Although the study was conducted during the time of Covid-19 emergence, Thailand had a strict policy to control the spread of the disease; therefore, there was minimal effect of Covid-19 on the patients’ initial participation in the study. However, some attrition of patients especially during the long-term follow-ups may have resulted due to the fear of pandemic spread among the population [14].

In comparison to a previous study on proactive screening of oral cancer in Roi Et province, Thailand [15], our study considered a larger target population. In the Roi Et study, 57,763 individuals were initially considered. VHVs distributed questionnaires to the subjects who answered the questions themselves and explored their own oral cavity. Only in the presence of suspicious oral lesions or risk factors, they were recruited for subsequent screening by dental auxiliaries at the sub-district hospital. A total of 2365 subjects were screened by them and 407 subjects were referred to the district hospitals. After the screening by dentists, 99 subjects were referred to the provincial hospital for final diagnosis and treatment. Eighty subjects (0.14%) attended this screening, among which 44 subjects exhibited OPMDs or oral cancer but only 10 subjects (0.017%) underwent biopsy. The histopathology results from Roi Et study indicated the presence of oral squamous cell carcinoma, epithelial dysplasia, lichen planus, and fibrous hyperplasia in 1, 2, 4, and 3 subjects, respectively [15]. One of the reasons for higher subject withdrawal (attrition rate of 77.3%) in the Roi Et study could have been due to the numerous screening steps. In comparison, our study started from 330,914 subjects which was approximately 5.7 times larger than the Roi Et study. A total of 1576 subjects (0.4%) attended the screening at district hospital and biopsy was performed by oral medicine specialists or oral surgeons in 427 subjects (0.11%).

In this study, the majority of subjects were female (Table 1). This was consistent with a study on the prevalence of oral premalignant lesions in Thai people conducted in Roi Et province by Juntanong et al. in which a female predilection was observed [12]. Greater awareness of oral health among women may be, in part, responsible for the likelihood to participate in the screening.

When considering the risk factors in each gender, a higher percentage of male subjects were smokers (42.3%) or ex-smokers (16.2%), alcohol drinkers (38.7%), and had regularly worked under sunlight (67.6%). Since the preponderance of the subjects in this study were farmers, their career would involve long durations of exposure to sunlight. On the contrary, higher percentage of female subjects had a history of betel quid chewing (56.0%) or smokeless tobacco use (18.5%). These results were in contrast to a field study by Reichart et al. conducted among the hill-tribe people of northern Thailand, in which a similar number of male and female subjects had the habit of betel quid chewing (15.8% vs. 18.9%) and tobacco use (15.8% vs. 17.4%) [16]. Although betel quid consumption has been declining in the general population, it is still persistent in some groups, especially in elderly people in the northeastern area of Thailand. An intercountry Asian Betel-quid Consortium study was conducted for Taiwan, Mainland China, Malaysia, Indonesia, Nepal, and Sri Lanka to investigate the prevalence, patterns of practice, and associated types of oral preneoplastic disorders [17]. A random group of 8922 subjects were recruited, and the data were analyzed using survey-data modules adjusted for the complex survey design. Chewing rates among men (10.7–43.6%) were significantly higher than women (1.8–34.9%) in Taiwan, Mainland China, Nepal, and Sri Lanka, while women’s rate (29.5–46.8%) were higher than that for men (9.8–12.0%) in Malaysia and Indonesia [17]. Since Thailand is in the same geographic region as Malaysia and Indonesia, a similar trend was observed as the prevalence of betel quid chewing habit among female subjects (56.04%) was higher than male (9.01%) in this study.

The oral lesions were categorized into three major types including oral cancer, OPMDs, and non-OPMDs/oral cancer (Tables 1 and 2). Oral cancer was observed in 25 specimens in 22 subjects. OPMDs accounted for 264 lesions in 236 subjects and the remaining 173 lesions in 151 subjects were non-OPMDs/oral cancer.

In the oral cancer category, squamous cell carcinoma and verrucous carcinoma were found in 14 and 6 specimens, respectively. While 5 specimens exhibited other types of cancers including 2 malignant salivary gland tumors, lymphoma, clear cell odontogenic carcinoma, and basal cell carcinoma. The incidence rate of oral cancer in this present study calculated by the estimation from target population was 5.9 per 100,000 subjects (22 subjects with positive oral and lip cancers in 371,911 subjects who were ≥ 40 years old). This incidence is comparable to the report by GLOBOCAN in 2020 where the world age-standardized incidence rate (ASR) for oral and lip cancers was 4.1 in 100,000 and the Thai ASR were 5.1 and 3.0 in 100,000 in male and female, respectively [1]. In the study from Roi Et province, oral squamous cell carcinoma was detected in 2 out of 57,763 target subjects (one confirmed by a biopsy and the other one by mere clinical diagnosis), and the incidence rate was 3.5 in 100,000 targeted population [15]. It is suggested that the incidence rate of cancer detected in this study was comparable to that of the world and Thailand as previously reported by GLOBOCAN 2020.

Oral leukoplakia is a defined as white plaque of questionable risk, having excluded (other) known diseases or disorders that carries no increased risk for cancer [9]. Oral leukoplakia, as well as OSCC is strongly associated with tobacco smoking and alcohol drinking, possibly not associated with HPV [18]. The histopathological biopsy results of oral leukoplakia include hyperkeratosis, acanthosis, epithelial dysplasia (mild, moderate, or severe), carcinoma in situ, and squamous cell carcinoma. Importantly, in this present study, white plaque and erythematous lesions were found predominantly during clinical examination which rendered the need for incisional or excisional biopsy. According to the histopathology results, the most prevalent histopathological diagnosis was mild epithelial dysplasia. It was advantageous as potentially malignant disorders were diagnosed more often than cancerous lesions. If these subjects are followed closely and the risk factors are reduced, the transformation to malignant oral lesions could be prevented. The lesions that presented with mild, moderate, or severe epithelial dysplasia were 126 (27.3%), 37 (8.0%), and 17 (3.7%) lesions, respectively. Overall, epithelial dysplasia was found in 180 (38.9%) specimens. In comparison, a study conducted by Lapthanasupkul at the Faculty of Dentistry, Mahidol University in 2007, analyzed 7177 biopsy specimens, in which 123 cases (1.7%) were oral leukoplakia [19]. Histopathologic study showed that 60.9% of the provisionally diagnosed cases with oral leukoplakia exhibited hyperkeratosis with or without acanthosis followed by lichen planus (11.4%), and only 10.6% were diagnosed as epithelial dysplasia. The subjects were recruited from the general population who may not have had any risk factors for oral cancer, whereas the subjects recruited for our study had at least one risk factor for oral cancer or OPMDs. In another study also conducted at the Faculty of Dentistry, Mahidol University in 2019, 130 OPMD and 78 squamous cell carcinoma cases that occurred at the tongue were retrospectively investigated [20]. Out of 130 specimens of OPMDs, 64 specimens were provisionally diagnosed as oral leukoplakia and 32 specimens (24.6%) exhibited mild epithelial dysplasia. Moderate, severe, and non-epithelial dysplasia accounted for 18.8%, 12.5%, and 18.8%, respectively [20].

The second most common oral lesion diagnosed from histopathology results was fibrous hyperplasia or fibroma which accounted for 14.5% (67 of 462 specimens). A large number of the subjects in this study had poor oral hygiene and did not have routine dental visits. Several of them had sharp teeth or irritation due to severe periodontal diseases, calculus deposition, or dental caries. In the study from Roi Et province, only 3 subjects had fibroma from histopathology analysis [15]. Irritation fibroma is a common lesion encountered in the elderly. A cross-sectional multicenter study conducted in 2016 investigated 11,346 biopsy records of subjects ≥ 65 years old from Canada, Iran, Japan, South Korea, and Thailand and revealed that focal fibrous hyperplasia or irritation fibroma was the second most common oral lesions found in the elderly, accounting for 9.7% [21]. Although irritation fibroma is not considered as an OPMD, it was interesting that dental auxiliaries could screen these abnormalities, including exophytic lesions in the oral cavity of the subjects, and referred them for further investigation.

The third most common oral lesion encountered was oral lichen planus/oral lichenoid reactions which accounted for 11.5% of the histopathology results (Table 3). Lichen planus is an immune-mediated disease with an unknown etiology, whereas oral lichenoid reactions could result from allergy to dental materials, medication, hepatitis C virus infection, or graft versus host disease. Since the majority of subjects in this study were elderly, they suffered from systemic diseases, including hypertension, hyperlipidemia, diabetes, joint problems, etc. Most of them were also on medications, such as antihypertensive drugs, sulfonylurea, simvastatin, and non-steroidal anti-inflammatory drugs (NSAIDs), which could induce oral lichenoid reactions. The clinical appearance of oral lichen planus/oral lichenoid reactions resembled that of oral leukoplakia and erythroplakia and required biopsy for definitive diagnosis. In fact, some of the cases with oral lichen planus/oral lichenoid reactions also displayed epithelial dysplasia; therefore, close follow ups of these subjects are necessary following treatment with topical steroids to ensure that the lesions are true oral lichen planus/oral lichenoid reactions and not lichenoid dysplasia. Furthermore, lichenoid lesion has been reported in association with betel nut or quid chewing habit and tobacco placement as well [22]. This lesion is exclusively revealed among betel quid users. The patterns of the lesion resemble that of lichen planus, but the white striations occur at the site of the betel quid placement with parallel patterns without any crisscross [20]. Moreover, this lesion can regress after habit cessation. Therefore, screened subjects were encouraged to quit all relevant risk factors.

Among the OPMD lesions, a female predominance was found. Subjects who used smokeless tobacco or had no history of oral cancer were also more likely to present with OPMDs. Moreover, in our analysis betel quid chewing was identified as a statistically significant risk factor associated with oral cancer and OPMDs (OR = 2.93, 95% CI 1.75–4.88) (Table 5). This was in accordance with a study conducted to investigate the associated risk factors of oral cancer in the northeastern area of Thailand in which betel quid chewing habit was associated with oral cancer in women (OR = 4.11, 95% CI 2.15–7.78) [23]. Longer durations of habit also increased the risk of oral cancer. Hence, in these subjects, reduction of these risk factors must be emphasized along with rigorous monitoring of malignant transformation to prevent the emergence of oral cancer.

There were also certain limitations in this study. Firstly, a high number of drop-outs were observed when subjects were asked to visit a hospital for oral  examination. In fact, when asked about traveling, they preferred to have someone visit their home and examine the oral cavity rather than going to the hospital themselves. In Thailand, the VHV system is an effective way of screening and providing care to the subjects who have risk factors for oral cancer in the local area. In Thailand, this system has been established since 1960s, and it is the backbone of the health care delivery system, supporting the concept of community involvement as the heart of public health care activities [24]. After the screening, the VHVs also assisted in the follow up of the subjects. With proper training on visual screening, we believe that early detection of suspicious oral lesions can be achieved by VHVs. Furthermore, they could also provide a sense of urgency and encourage the subjects to visit the hospital. Moreover, the cost-effectiveness of the program can also be significantly increased, if the subjects themselves could screen their own oral cavity for abnormalities. High sensitivity and specificity of the visual examination by trained dental nurses and dentists must also be achieved, so that the program can be more successful and cost-effective. However, even in well-structured population screening programs, dental professionals may encounter difficulties to recognize lesions at risk; thus, necessitating referrals to specialized care, such as auxiliary methods and fluorescence visualization to confirm the diagnosis [25, 26]. Furthermore, Oral Brush Biopsy has been demonstrated as a great tool for oral cancer–finding and surveillance programs [25].

Secondly, in this study patients ≥ 40 years old were the target population, even though OSCC and OPMDs are known to occur in young patients. Although some authors consider head and neck squamous cell carcinoma as a particular entity, most studies demonstrate that population younger than 40 years old is not as frequently affected by HNSCC as the population older than 40 years old. However, tobacco smoking and alcohol drinking remain as great risk factors for HNSCC in the young population [27]. It is unclear whether the results would be different if we did not include age ≥ 40 years old as one of the inclusion criteria. Future studies may still be needed to clarify this point.

## Conclusions

In this study, the five most common histopathology results of oral cancer and OPMDs screening in the subjects from the northeastern Thailand were mild epithelial dysplasia, fibro-epithelial hyperplasia, oral lichen planus/oral lichenoid reactions, moderate epithelial dysplasia, and acanthosis with or without hyperkeratosis. Habitual betel quid chewing was established as a significant risk factor for OPMDs and oral cancer in this study population. Therefore, rigorous monitoring of the subjects with OPMDs are encouraged to prevent oral malignant transformation.

## Supplementary Information

**Additional file 1**. Distribution of risk factors of oral cancer according to types of oral lesions.

## Data Availability

The datasets used and/or analyzed during the current study are not publicly available due the confidentiality of the participants but are available from the corresponding author on reasonable request.

## Abbreviations

OPMDs: Oral potentially malignant disorders
VHVs: Village healthcare volunteers
S1: Screening 1
S2: Screening 2
S3: Screening 3
GLOBOCAN: Global cancer observatory
CI: Confidence interval
OR: Odds ratio
SD: Standard deviation
GVHD: Graft versus host disease
WHO: World Health Organization
NSAIDs: Non-steroidal anti-inflammatory drug
