# Supplementary material for: Histopathological and risk factor analyses of oral potentially malignant disorders and oral cancer in a proactive screening in northeastern Thailand
Source: BMC Oral Health. 2022 Dec 16;22:613. doi: 10.1186/s12903-022-02646-9 (PMC9756922; doi:10.1186/s12903-022-02646-9)
Supplement: Supplementary file 1 — Additional file 1. Distribution of risk factors of oral cancer according to types of oral lesions. [file 12903_2022_2646_MOESM1_ESM.pdf]

**Distribution of risk factors of oral cancer according to types of oral lesions.**

| Characteristics | Non-OPMD/cancer | | OPMD* | | Cancer | | *p*-value |
|---|---|---|---|---|---|---|---|
| | n | % | n | % | n | % | |
| **Sex (n = 409)** | | | | | | | |
| Male | 49 | 32.45 | 53 | 22.46 | 9 | 40.91 | 0.032 |
| Female | 102 | 67.55 | 183 | 77.54 | 13 | 59.09 | |
| **Age (n=409)** | | | | | | | |
| 40-49 | 10 | 6.62 | 8 | 3.39 | 1 | 4.55 | 0.088 |
| 50-59 | 35 | 23.18 | 37 | 15.68 | 6 | 27.27 | |
| 60-69 | 55 | 36.42 | 93 | 39.41 | 4 | 18.18 | |
| 70-79 | 41 | 27.15 | 82 | 34.75 | 7 | 31.82 | |
| 80-89 | 10 | 6.62 | 13 | 5.51 | 3 | 13.64 | |
| ≥90 | 0 | 0.00 | 3 | 1.27 | 1 | 4.55 | |
| Mean ± SD* | 64.64 ± 9.59 | | 67.19 ± 9.08 | | 69.00 ± 12.74 | | |
| Minimum-maximum | 42-89 | | 43-91 | | 48-94 | | |
| **Smoking (n = 409)** | | | | | | | |
| Never | 122 | 80.79 | 196 | 83.05 | 17 | 77.27 | 0.089 |
| Smoker | 16 | 10.60 | 32 | 13.56 | 5 | 22.73 | |
| Ex-smoker | 13 | 8.61 | 8 | 3.39 | 0 | 0.00 | |
| **Smokeless tobacco (n= 404)** | | | | | | | |
| Never | 132 | 88.00 | 177 | 75.64 | 17 | 85.00 | 0.012 |
| Smoker | 13 | 8.67 | 51 | 21.79 | 3 | 15.00 | |
| Ex-smoker | 5 | 3.33 | 6 | 2.56 | 0 | 0.00 | |
| **Secondhand smoker (n = 386)** | | | | | | | |
| No | 104 | 73.24 | 157 | 69.78 | 12 | 63.16 | 0.707 |
| Yes | 38 | 26.76 | 68 | 30.22 | 7 | 36.84 | |
| **Alcohol drinker (n = 406)** | | | | | | | |
| Never | 100 | 66.67 | 174 | 74.36 | 14 | 63.64 | 0.345 |
| Alcohol drinker | 37 | 24.67 | 40 | 17.09 | 6 | 27.27 | |
| Ex-alcohol drinker | 13 | 8.67 | 20 | 8.55 | 2 | 9.09 | |
| **Betel quid chewing habit (n = 408)** | | | | | | | |
| Never | 104 | 69.33 | 94 | 39.83 | 13 | 59.09 | <0.001 |
| Chewer | 43 | 28.67 | 127 | 53.81 | 7 | 31.82 | |
| Ex-chewer | 3 | 2.00 | 15 | 6.36 | 2 | 9.09 | |
| **Working in sunlight (n = 404)** | | | | | | | |
| No | 67 | 45.27 | 108 | 46.15 | 12 | 54.55 | 0.717 |
| Yes | 81 | 54.73 | 126 | 53.85 | 10 | 45.45 | |
| **History of head and neck cancers (n = 404)** | | | | | | | |
| No | 133 | 90.48 | 228 | 97.02 | 19 | 86.36 | 0.009 |
| Yes | 14 | 9.52 | 7 | 2.98 | 3 | 13.64 | |

*OPMD: oral potentially malignant disorders, SD: standard deviation
